# Knowledge, Preference, and Willingness to Pay for Hepatitis B Vaccination Services among Woman of Reproductive Age in Vietnam

**DOI:** 10.1155/2019/9154918

**Published:** 2019-02-21

**Authors:** Anh Tuan Le Nguyen, Xuan Thanh Thi Le, Toan Thanh Thi Do, Cuong Tat Nguyen, Long Hoang Nguyen, Bach Xuan Tran, Huong Thi Le

**Affiliations:** ^1^Institute for Preventive Medicine and Public Health, Hanoi Medical University, Hanoi, Vietnam; ^2^Institute for Global Health Innovations, Duy Tan University, Da Nang, Vietnam; ^3^Center of Excellence in Behavioral Medicine, Nguyen Tat Thanh University, Ho Chi Minh City, Vietnam

## Abstract

**Background:**

Hepatitis B virus (HBV) vaccine is a critical approach to prevent HBV transmission from mother to child. However, despite high HBV prevalence, evidence about the preference of women of productive age for HBV vaccine in Vietnam was constrained. This study aims to explore the preference and willingness to pay (WTP) for the HBV vaccine in Vietnamese women in productive age.

**Methods:**

A cross-sectional study was conducted in Hanoi in April 2016. A structured questionnaire was used to collect information about respondents' socioeconomic status and knowledge about HBV vaccination. A contingent valuation approach was employed to measure the WTP for the HBV vaccine. Logistic and interval regressions were used to determine the associated factors.

**Results:**

Among 807 women, 80.8% were willing to have the vaccine injected which had the average price of 108,600 VND (95% CI, 97,580 VND–119,570 VND). Participants not suffering any diseases during pregnancy were more likely to be willing to pay for the HBV vaccine (OR = 3.41, 95% CI = 1.73–6.70). Not having the antenatal examination at central hospitals and working as farmers/workers were positively correlated with willingness to pay for this vaccine, while the number of children of respondents had a negative correlation with WTP.

**Conclusions:**

Our sampled women expressed a high willingness to pay for the vaccine. The price people were willing to pay for the vaccine, however, is equal to half of the actual price. These findings implied needs for better targeted public education interventions about HBV and the involvement of local medical staffs and the media in providing information. Efforts to reduce the price of the vaccine should also be warranted for scaling-up the coverage of this vaccine.

## 1. Introduction

Hepatitis B (HBV) is a viral infection that has been well-recognized as a serious global health concern [1]. The World Health Organization (WHO) estimated its death tolls to be about 887,000 per year, while roughly 257 million people worldwide are currently disease carriers [2]. Vietnam is an HBV epidemic country as the prevalence of positive HB surface antigen (HbsAg+) in two metropolitans ranges from 9 to 14% [3]. A recent study forecasted that the number of chronic HBV cases in Vietnam will amount to 8 million by 2025 with HBV–related annual mortality reaching 40,000 [4].

One of the major HBV transmission routes is vertical transmission or mother-to-child [5]. In countries where HBV is an endemic problem, it was estimated that 50% of the HBV positive cases got their infections from either perinatal or early childhood; the chance of such infection developing into chronic Hepatitis B was 70% to 90% [6, 7]. Even when most young children were sufficiently HBV vaccinated, the mother-to-child transmission still resulted in a 40% to 50% of the new HBV positive cases [5]. Thus, effective prevention of this vertical HBV transmission, especially through treating the potentially infected mothers and would-be mothers, can be said to be crucial in decreasing the burden of HBV.

Current vaccines were found to be remarkably effective against chronic HBV infection with the rate of prevention ranging from 94 to 98% [8]. However, limited success has been reported for the vaccination program targeting women of reproductive ages, due to a number of constraints [9]. Some studies have showed that insufficient attendance of antenatal care and poor knowledge on vaccinating pregnant women would have adverse impact on the efficiency of maternal health care providers in developing countries, while others pointed to a shortage of practical knowledge about immunization programs in younger, poorly educated, and illiterate mothers [10, 11]. Access barriers to the vaccination program and a lack of adherence to standard infection control precautions have also been possible causes [12].

Despite high HBV prevalence, researches looking into HBV prevention in women of productive age in Vietnam have been scarce. This study attempts to partially fill this gap in the literature, exploring several aspects of HBV vaccination in Vietnamese mothers: their awareness of HBV vaccine, willingness to be vaccinated and to communicate about vaccination, and potential influencing factors, employing a contingent valuation approach.

## 2. Material and Methods

### 2.1. Survey Design and Sampling Procedure

A cross-sectional study was conducted in two districts of Hanoi, including the Dong Da district and Ba Vi district from April 1 to April 30, 2016. Two communes in each district were selected for the survey. In Dong Da, Trung Tu commune and Phuong Lien commune were selected. In Ba Vi, Thuy An commune and Phong Van commune were selected. The subjects of the survey were women living in the selected site. The eligibility criteria also included (1) being pregnant or having children under 1 year of age; (2) living at research site for at least 6 months; (3) agreeing to join the survey; (4) not suffering from HBV disease before.

A formula to estimate a population proportion with specified absolute precision was used to calculate the sample size. With the expected proportion of women being willing to pay for HBV vaccine = 0.5 (for maximizing the sample size) and absolute precision d = 0.07, the sample for each commune was 196 women. We added 10% for compensation rate; the final sample size per commune was 216 women. We listed all women who met our inclusion criteria in each commune with the support of local authorities. Then, we randomly selected participants using computer software. “If one individual did not accept to participate, we selected the one who was next to them in the list. Detailed information was presented in Table 1.

### 2.2. Measures and Instruments

We conducted face-to-face interviews by well-trained staffs and students from Ba Vi district health center and Hanoi Medical University. A structured questionnaire was used to gather data about respondents' socioeconomic status and knowledge about and willingness to pay for Hepatitis B vaccination.

#### 2.2.1. Socioeconomic Information and Self-Rated Health

Data about age, pregnancy status, occupation, education, internet usage, and health facilities in which the antenatal examination took place and average income were self-reported. Self-rated health status was also collected by asking participants to rate their health in four categories: “Very good,” “Good,”,“Normal,” and “Weak.”

#### 2.2.2. Source of Vaccine Information

The indicators to measure the source of vaccine information were as follows: information resources, the types of vaccine information that respondents wanted to know, preference on the channel of communication about vaccination, and the awareness of Hepatitis B vaccine price.

#### 2.2.3. Willingness to Have Hepatitis B Vaccine Injected

**Figure 1:**
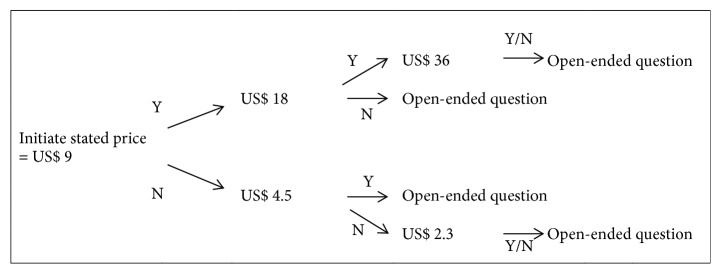
Bidding process.

To elicit willingness to have Hepatitis B vaccine injected, double-bounded dichotomous-choice questions were utilized to ask the respondents whether they were willing to be vaccinated. In this study, 200,000 VND (~ US$ 9, 2016 exchange rate) was used as a starting bid based on the actual price of this vaccine in the clinics. Firstly, they were asked whether they were willing to pay 200,000 VND per vaccine injection, and then they would move to the double bid for “Yes” response and the half bid for “No” response. Finally, the respondents had to answer an open-ended question about the maximum price they would be willing to pay to have Hepatitis B vaccine injected. The bidding process was described in Figure 1.

### 2.3. Statistical Analysis

The descriptive statistical analysis was used to describe the sociodemographic characteristics and knowledge about HBV vaccine of respondents (including information resources, the types of vaccine information that respondents wanted to know about, and preference on the channel of communication about vaccination). The significance level was set at p < 0.05. Logistic regression was performed to determine the factors related to willingness to get vaccinated and communicate about Hepatitis B vaccination, combined with a backward stepwise selection strategy. Interval regression was used to measure the amount of WTP.

### 2.4. Ethical Approval

The study protocol was approved by the Institutional Review Board (IRB) of Hanoi Medical University. The data collection at study sites was approved and supported by Dong Da and Ba Vi District Health Centre. Written informed consent was obtained from all participants after clearly introducing the survey. Respondents were informed that they could refuse to participate or withdraw from the study at any time they want.

## 3. Results

Majority of participants were having a baby under 1 year of age (76%), over 25 years old (82.6%), having finished high school or higher education (82.7%), working as workers/farmers/public servants (61.8%), and reportedly in good health (64.2%). Private/international hospital was the most popular place of choice among respondents for having antenatal examination (35.3%), followed by central hospital (28.9%). The average number of the antenatal examination was 8 times (Table 2).

91.5% of respondents have heard about Hepatitis B vaccine, mostly via television (55.6%) and from medical staffs (55.5%). Most of the participants wanted to communicate about the vaccine (89.7%), with three most commonly requested topics of information being benefits of vaccine (64.8%), schedules of vaccination (37.5%), and consequences of nonvaccination (32.8%). A most preferred channel of communication was the health worker's advice (29.3%). 87.3% of those interviewed were not aware of the vaccine price (Table 3).

Among participants, 80.8% were willing to have the vaccine injected which had the average price of 108,600 VND (SD=142,700). Willingness to vaccinate was highest in farmers (93.2% of them would accept vaccination), people under 25 years old (90.3%), those with an education level of high school and lower (83.0%-88.9%), and those without health insurance (82.8%). However, willingness to pay was the lowest in these exact subgroups, with willingness to pay the average prices of 79,000 VND; 87,000 VND; 73,100–100,700 VND; 87,400 VND, respectively. Meanwhile, business managers were willing to pay the highest price for the vaccine (197,000 VND; SD=246,200) (Table 4).

Participants who did not suffer from the disease during pregnancy were more likely to demand vaccination (OR = 3.41, 95% CI = 1.73–6.70). Not having the antenatal examination at central hospitals and working as farmers/workers were positively correlated with willingness to be vaccinated, while the number of children of respondents displayed a negative correlation with willingness. People who had the antenatal examination in places other than central or private/international hospitals were more likely to want more information about the vaccine, whereas those working as public servants or currently in good health were less likely to demand communication about a vaccine, compared to stay-at-home mothers and people in very good health status. The number of children of respondents was also negatively associated with the willingness to communicate (Table 5).

## 4. Discussion

This study found that despite a high rate of awareness of HBV vaccine, a majority of participants were still willing to know more about the vaccine, especially about its benefits. Willingness to be vaccinated was generally high; however, the average price respondents were willing to pay for vaccination was just half of the offered price. Sociodemographic characteristics of those interviewed were found to influence their preferences toward vaccination.

Although a majority of respondents were found to know about HBV vaccine, their more in-depth knowledge regarding the vaccine may still be lacking, as most of them were willing to acquire rather basic information such as the vaccine's benefits. This finding is comparable to a study on Chinese pregnant women that reported the high rate of HBV vaccine awareness (65%-92% of respondents knew HBV could be prevented by vaccination) but low rate of awareness on HBV transmission mechanism [13]. Other studies on women with children in other developing countries reported a substantially lower level of HBV knowledge; 34.7% of those were interviewed in Ghana and 12.2% in Kenya [14, 15]. Thus, it can be said that in Vietnam, some success has been achieved in communication about the disease and vaccine to the public, especially via mass media. According to the study, television was one of the most common channels from which information about the HBV vaccine was obtained. On the other hand, health workers' advice was the most preferred vaccination communication channel, probably due to the higher level of trust people generally have for health workers regarding health topics, compared to other sources of information. This highlighted the importance of medical staffs, especially those at a local level and more remote areas, in providing sufficient knowledge of diseases and cures to the community [16].

The high rate of willingness to take HBV vaccination discovered in the study was in line with the finding from a Ghana study showing that 93.8% of pregnant women surveyed would take medication to prevent HBV [17]. In contrast, a Chinese study stated that only 16.5% of participants would accept HBV preventive drugs during pregnancy [18]. Lack of HBV knowledge, which lead to, among others, unnecessary worry about the safeness of the vaccine to the child, was cited by the author of that study as one of the causes of such low willingness level. Our study found instead that people not being examined at central hospitals and who have enjoyed healthy pregnancy were more willing to have the vaccine injected, which indicated a level of trust participants have in preventive medication. Such a view was shared by a study by Guo Na et al (2017) in China which found both living in urban areas and having higher income level would likely mean better knowledge on HBV vaccine and were found to be positively correlated with the acceptance to get vaccinated [19].

The average price that respondents of this study deemed affordable for HBV vaccine was lower than clinics' listed price, though the willingness to pay varied among different subgroups. Younger mothers without jobs or with lower paying ones and those without health insurance were willing to pay just about a third of the offered price. Such low willingness to pay may be due to the financial difficulties of these participants but may also be the result of insufficient knowledge of the danger of the disease and benefits of getting vaccinated. Researches had indeed found a correlation between lack of comprehensive understanding regarding the importance of vaccination and low willingness to pay for the vaccine in adults [20]. Nonetheless, low willingness to pay would pose a threat to increase the coverage of HBV vaccination in Vietnam. Lowering the vaccine price would be immensely challenging in Vietnam context, one may argue, as the price currently offered has already reflected subsidizations benefited from the support of Global Alliance for Vaccines and Immunization (GAVI) [21]. Therefore, providing the public with appropriate information on the disease and life-saving benefits of vaccination would be a more probable and effective solution.

In this study, we found that women with lower levels of education had high rate of willingness to vaccinate, but this association was not statistically significant when being adjusted in regression model. Previous studies in China were in line with us when it was found that education was negatively correlated with the need of HBV vaccine and willingness to pay for it [16, 22]. The reason of this phenomenon is unclear. However it may be explained by the lack of familiarity with HBV vaccines among participants with low education, or in other words, the percentage of people being aware of HBV vaccine was higher among those with higher education level (data not shown). Indeed, contingent valuation method uses hypothetical scenarios to describe HBV vaccine that would be offered in the future; thus, participants may not know about the HBV vaccine before hearing from interviewers. In literature, insufficient familiarity with the proposed HBV vaccines could result in hypothetical bias that may lead to overestimating participants' WTP [23].

The current study also indicated that people who were farmers/workers were more likely to be willing to pay for the vaccination. Farmers/workers are among those who are most at risk of HBV infection. A study in Iran revealed that HBV prevalence among farmers was the highest compared to other occupations [24]. Another nationwide study in Lao PDR depicted that 71% of HBV infected mothers were farmers [25]. Therefore, we assumed that farmers/workers in our study were aware of their risk and more likely to be willing to pay for the vaccine than other people.

Few implications can be drawn from this study. Education campaigns covering topics of HBV transmission mechanism, especially from mother-to-child, essentialism of vaccination, and how paying to get vaccinated would work as a cost-effective solution with lifelong effect should be developed and ran more frequently, targeting younger and poorer women of reproductive age. Media should be continuously used as the main channel via which information reaches the public, while the active participation of medical staffs at the less central level in this educational effort should be encouraged. In addition, although it seems challenging, efforts should be made by the government to look for ways of reducing the vaccine price, possibly through encouraging additional contribution from the private sector via corporation social responsibility program or from philanthropists. The government also should enforce the coverage of health insurance, especially to the lesser fortunate group.

This study has several limitations. Its cross-sectional, self-reported setting would allow only a ‘snap-shot' of information at the time of study with potential inaccuracy resulting from recalling errors of the participant. Though efforts were made to include a relatively large number of respondents from diverse backgrounds, the sample of this study cannot be said to be representative of the population concerned. Moreover, further researches are encouraged to incorporate the rate of vaccination among participants or explore the influencing factors of respondents' willingness to pay for the vaccine which are topics that had not been covered in this study.

## 5. Conclusion

The study aimed to explore several aspects of HBV vaccination among woman of reproductive age in Vietnam. Although those interviewed demonstrated high awareness of the vaccine's existence, further knowledge regarding the benefits and price of the vaccine was limited. Participants however expressed high willingness to communicate about the disease as well as to be vaccinated. The price people were willing to pay for the vaccine, however, was on average just half of the often-quoted price. These findings implied the need for better targeted public education regarding the danger and solution of HBV, active participation from local medical staffs and the media in providing information, and efforts to reduce the listed price of the vaccine.

## Figures and Tables

**Table 1:** Study settings and sample size.

Level	Settings	Total woman fitting	Sample size
		the research criteria	
District (rural)	Phu Son Commune	220	207
	Thuy An Commune	200	200
District (urban)	Trung Tu Commune	465	200
	Phuong Lien Commune	410	200

**Table 2:** Demographics of respondents.

Characteristics	*N*	%
*Pregnancy status*		
Pregnant	190	24.0
Having a baby under 1 year of age	602	76.0
*Age group*		
Under 25 years old	138	17.5
From 25 to 30 years old	374	47.3
Above 30 years old	279	35.3
*Education attainment*		
Lower Secondary school	137	17.3
High school	230	29.2
College	178	22.6
University and above	244	30.9
*Occupation*		
Homemaker	108	13.7
Farmer/ Workers	267	33.8
Public servants	221	28.0
Others	194	24.6
*Facilities where having an antenatal examination*		
Commune/ward medical station	174	21.9
District/province hospital	197	24.8
Central hospital	229	28.9
Private/International hospital	280	35.3
Others	15	1.9
No Answer	76	9.6
*Current health status*		
Very good	31	4.4
Good	455	64.2
Normal	244	34.4
Weak	10	1.4
*Suffered from disease during pregnancy*	58	7.4

	Mean	SD

*The number of antenatal examination*	8.0	4.7
*Average Income per month (mil VND)*	12.8	9.3
*The number of children of the respondent*	1.7	0.8

**Table 3:** The knowledge about HBV vaccine of respondents.

	*N*	%
*Heard about Hepatitis B vaccine*	*720*	*91.5*
*Information resources*		
At school	40	5.0
Via television	441	55.6
Via listening radio	160	20.2
By reading a newspaper, magazines	174	21.9
Internet	323	40.7
Medical staff	440	55.5
Friends, relatives	177	22.3
*Communication about vaccine*	*695*	*89.7*
*The information of the vaccine they want to know*		
Benefits of vaccine	514	64.8
Schedules of vaccine	297	37.5
Vaccine location	158	19.9
Consequences on non-vaccination	260	32.8
Free-of-charge vaccines and location	145	18.3
The price of vaccines	217	27.4
The type of vaccines	226	28.5
Other	33	4.2
*Preference on a channel of communication about vaccination*		
TVs	116	14.9
Radios	73	9.4
Newspaper/magazine	16	2.1
Posters/ Leaflets	26	3.3
Cell phone	44	5.7
Health worker's advice	228	29.3
A guideline in the vaccination booklet	29	3.7
Direct talks	98	12.6
Integrate with local meetings	9	1.2
Internet	112	14.4
Other	11	1.4
*Know the price of hepatitis B vaccine*		
Yes	47	6.0
No	690	87.3
Free	53	6.7

**Table 4:** The willingness to be vaccinated with Hepatitis B vaccine.

	N	Willingness to		Price of willingness to	
		vaccinate		pay for the vaccine	
		N	%	Mean	SD
*Total*	*805*	*650*	*80.8*	*108.6*	142.7
*Occupation*					
Homemaker	110	87	79.1	87.9	108.1
Farmer	221	206	93.2	79.1	83.3
Public servants	222	169	76.1	144.3	171.2
Workers	57	49	86.0	84.3	139.5
Self-Business	136	95	69.9	103.5	119.1
Business Manager	9	7	77.8	197.0	246.2
Others	47	34	72.3	176.0	240.6
*Age group*					
Under 25 years old	144	130	90.3	87.0	106.1
From 25 to 30 years old	376	304	80.9	117.3	151.6
Above 30 years old	283	215	76.0	109.2	145.9
*Education attainment*					
Illiteracy/Primary	27	24	88.9	100.7	76.0
Secondary school	116	103	88.8	87.7	101.1
High school	235	195	83.0	73.1	92.3
College	180	142	78.9	102.9	127.5
University	220	166	75.5	162.6	201.4
Post graduated	23	16	69.6	145.9	149.5
*Having Health insurance*					
Yes	508	403	79.3	122.6	161.6
No	290	240	82.8	87.4	105.0

**Table 5:** Factor associated with willingness to inject and communicate about Hepatitis B vaccination.

	Willingness to have Hepatitis B		Wanting to communicate about	
	vaccines injected		vaccines	
	OR	95% CI	OR	95% CI
*Pregnant / Having a baby under 1 year of age (Vs. Yes)*	1.94*∗∗*	(1.11 - 3.38)	2.07*∗*	(0.96 - 4.48)
*Health facilities where having the antenatal examination *				
Central hospital (No vs. Yes)	3.21*∗∗∗*	(1.99 - 5.18)	2.39*∗∗∗*	(1.30 - 4.40)
Private/International hospital (No vs. Yes)	1.47*∗*	(0.94 - 2.31)	2.30*∗∗∗*	(1.30 - 4.09)
*Current health status (Ref - Very good)*				
Good	1.50*∗*	(0.98 - 2.30)	0.39*∗∗∗*	(0.22 - 0.72)
Week	5.36	(0.60 - 48.00)		
*Suffered from disease during pregnancy (No vs. Yes)*	3.41*∗∗∗*	(1.73 - 6.70)		
*Occupations (Ref - Homemaker)*				
Farmer/ Worker	2.63*∗∗∗*	(1.47 - 4.73)		
Public servants	1.45	(0.89 - 2.34)	0.26*∗∗∗*	(0.13 - 0.55)
Others			0.48*∗*	(0.22 - 1.03)
*The number of children of respondent*	0.68*∗∗∗*	(0.52 - 0.91)	0.55*∗∗∗*	(0.39 - 0.79)
*Constant*	0.33*∗∗*	(0.12 - 0.93)	19.09*∗∗∗*	(5.73 - 63.56)

*∗∗∗* <0.01, *∗∗* <0.05, *∗* <0.1

## Data Availability

The data used to support the findings of this study are available from the corresponding author upon request.
